# Increased monocyte count as a cellular biomarker for poor outcomes in fibrotic diseases: a retrospective, multicentre cohort study

**DOI:** 10.1016/S2213-2600(18)30508-3

**Published:** 2019-06

**Authors:** Madeleine K D Scott, Katie Quinn, Qin Li, Robert Carroll, Hayley Warsinske, Francesco Vallania, Shirley Chen, Mary A Carns, Kathleen Aren, Jiehuan Sun, Kimberly Koloms, Jungwha Lee, Jessika Baral, Jonathan Kropski, Hongyu Zhao, Erica Herzog, Fernando J Martinez, Bethany B Moore, Monique Hinchcliff, Joshua Denny, Naftali Kaminski, Jose D Herazo-Maya, Nigam H Shah, Purvesh Khatri

**Affiliations:** a Institute for Immunity, Transplantation, and Infection, Stanford University School of Medicine, Stanford, CA, USA; b Division for Biomedical Informatics Research, Department of Medicine, Stanford University School of Medicine, Stanford, CA, USA; c Department of Biophysics, Stanford University School of Medicine, Stanford, CA, USA; d Section of Pulmonary, Critical Care, and Sleep Medicine, Department of Medicine, Yale School of Medicine, Yale University, New Haven, CT, USA; e Department of Biomedical Informatics, Vanderbilt University Medical Center, Nashville, TN, USA; f Department of Medicine, Division of Rheumatology, Northwestern University Feinberg School of Medicine, Chicago, IL, USA; g Division of Allergy, Pulmonary and Critical Care Medicine, Department of Medicine, Vanderbilt University School of Medicine, Nashville, TN, USA; h Department of Biostatistics, Yale School of Public Health, Yale University, New Haven, CT, USA; i Department of Medicine, Weill Cornell Medical College, Cornell University, New York, NY, USA; j Department of Internal Medicine, University of Michigan, Ann Arbor, MI, USA

## Abstract

**Background:**

There is an urgent need for biomarkers to better stratify patients with idiopathic pulmonary fibrosis by risk for lung transplantation allocation who have the same clinical presentation. We aimed to investigate whether a specific immune cell type from patients with idiopathic pulmonary fibrosis could identify those at higher risk of poor outcomes. We then sought to validate our findings using cytometry and electronic health records.

**Methods:**

We first did a discovery analysis with transcriptome data from the Gene Expression Omnibus at the National Center for Biotechnology Information for 120 peripheral blood mononuclear cell (PBMC) samples of patients with idiopathic pulmonary fibrosis. We estimated percentages of 13 immune cell types using statistical deconvolution, and investigated the association of these cell types with transplant-free survival. We validated these results using PBMC samples from patients with idiopathic pulmonary fibrosis in two independent cohorts (COMET and Yale). COMET profiled monocyte counts in 45 patients with idiopathic pulmonary fibrosis from March 12, 2010, to March 10, 2011, using flow cytometry; we tested if increased monocyte count was associated with the primary outcome of disease progression. In the Yale cohort, 15 patients with idiopathic pulmonary fibrosis (with five healthy controls) were classed as high risk or low risk from April 28, 2014, to Aug 20, 2015, using a 52-gene signature, and we assessed whether monocyte percentage (measured by cytometry by time of flight) was higher in high-risk patients. We then examined complete blood count values in the electronic health records (EHR) of 45 068 patients with idiopathic pulmonary fibrosis, systemic sclerosis, hypertrophic cardiomyopathy, or myelofibrosis from Stanford (Jan 01, 2008, to Dec 31, 2015), Northwestern (Feb 15, 2001 to July 31, 2017), Vanderbilt (Jan 01, 2008, to Dec 31, 2016), and Optum Clinformatics DataMart (Jan 01, 2004, to Dec 31, 2016) cohorts, and examined whether absolute monocyte counts of 0·95 K/μL or greater were associated with all-cause mortality in these patients.

**Findings:**

In the discovery analysis, estimated CD14+ classical monocyte percentages above the mean were associated with shorter transplant-free survival times (hazard ratio [HR] 1·82, 95% CI 1·05–3·14), whereas higher percentages of T cells and B cells were not (0·97, 0·59–1·66; and 0·78, 0·45–1·34 respectively). In two validation cohorts (COMET trial and the Yale cohort), patients with higher monocyte counts were at higher risk for poor outcomes (COMET Wilcoxon p=0·025; Yale Wilcoxon p=0·049). Monocyte counts of 0·95 K/μL or greater were associated with mortality after adjusting for forced vital capacity (HR 2·47, 95% CI 1·48–4·15; p=0·0063), and the gender, age, and physiology index (HR 2·06, 95% CI 1·22–3·47; p=0·0068) across the COMET, Stanford, and Northwestern datasets). Analysis of medical records of 7459 patients with idiopathic pulmonary fibrosis showed that patients with monocyte counts of 0·95 K/μL or greater were at increased risk of mortality with lung transplantation as a censoring event, after adjusting for age at diagnosis and sex (Stanford HR=2·30, 95% CI 0·94–5·63; Vanderbilt 1·52, 1·21–1·89; Optum 1·74, 1·33–2·27). Likewise, higher absolute monocyte count was associated with shortened survival in patients with hypertrophic cardiomyopathy across all three cohorts, and in patients with systemic sclerosis or myelofibrosis in two of the three cohorts.

**Interpretation:**

Monocyte count could be incorporated into the clinical assessment of patients with idiopathic pulmonary fibrosis and other fibrotic disorders. Further investigation into the mechanistic role of monocytes in fibrosis might lead to insights that assist the development of new therapies.

**Funding:**

Bill & Melinda Gates Foundation, US National Institute of Allergy and Infectious Diseases, and US National Library of Medicine.

## Introduction

Fibrotic diseases are estimated to contribute to up to 45% of all deaths every year in the USA,^1^ with the prognosis for many of them similar to that of terminal cancer.^2^, ^3^ There are very few treatment options for a patient with a fibrotic disease, and the only treatment with definitive evidence to prolong lifespan in fibrotic diseases of the lung such as idiopathic pulmonary fibrosis is a lung transplantation.^4^ Idiopathic pulmonary fibrosis is currently the most common indication for a lung transplantation in the USA.^5^

**Research in context**

**Evidence before this study**

New metrics are needed to identify high-risk patients with idiopathic pulmonary fibrosis who present with similar clinical variables as low-risk patients. We searched PubMed for articles using the terms “pulmonary fibrosis” AND “biomarkers” AND “blood”, with no date restrictions and not limited to English-language publications. We identified multiple studies that investigated proteins and genes in the blood associated with poor outcomes in patients with idiopathic pulmonary fibrosis. We found one study that prospectively validated a 52-gene peripheral blood gene signature for identifying high-risk patients with idiopathic pulmonary fibrosis in independent cohorts, but a substantial amount of work is required before this signature can be translated into clinical practice. We found no studies that identified and validated a cellular prognostic biomarker for idiopathic pulmonary fibrosis.

**Added value of this study**

In our study, we showed that increased monocyte count is associated with increased risk of poor outcomes in patients with idiopathic pulmonary fibrosis. We validated this association in more than 7000 patients with idiopathic pulmonary fibrosis from five independent cohorts. After correcting for gender, age, and disease severity metrics, increased monocyte count continued to be associated with poor outcomes in patients with idiopathic pulmonary fibrosis. Finally, we showed that higher absolute monocyte count at the time of diagnosis is also a prognostic marker of mortality in patients with other fibrotic diseases such as hypertrophic cardiomyopathy, systemic sclerosis, and myelofibrosis.

**Implications of all the available evidence**

Absolute monocyte count at the time of diagnosis is a reproducible prognostic marker of poor outcomes in idiopathic pulmonary fibrosis and other fibrotic diseases. These results suggest that monocyte count should be incorporated into the clinical assessment of patients with idiopathic pulmonary fibrosis and other fibrotic diseases. Absolute monocyte count is a component of a complete blood count, a widespread and globally available routine clinical test, making this biomarker inexpensive and easy to implement. Further investigation into the mechanistic role of monocytes in fibrosis might lead to insights that assist the development of new therapies.

Disease severity metrics are used to stratify patients with lung fibrosis for transplantation allocation. For idiopathic pulmonary fibrosis, disease severity is determined by age, sex, and pulmonary function combined into a composite index, such as the gender, age, physiology (GAP) index.^6^, ^7^ However, many patients present with similar clinical variables at the time of diagnosis.^7^, ^8^, ^9^ Furthermore, change in lung function is a better predictor of mortality than baseline lung function, which requires follow-up at 6 months.^10^, ^11^ Additionally, idiopathic pulmonary fibrosis can follow an unpredictable clinical course, with sudden decreases in lung function following periods of disease stability (eg, an acute exacerbation due to occult infection).^12^ Similarly, forced vital capacity (FVC) values are often misleading in idiopathic pulmonary fibrosis because of the confounding effects of concurrent emphysema.^13^ These issues highlight the need for additional prognostic metrics, which has led to efforts to define molecular signatures for identifying patients with idiopathic pulmonary fibrosis at high risk of mortality.^9^, ^14^, ^15^, ^16^, ^17^, ^18^ Although one 52-gene signature for identifying patients with a high risk of mortality has been validated in prospective cohorts,^19^ a substantial amount of work is needed before a signature can be translated into clinical practice. The identification and validation of an easily measured prognostic biomarker has the potential to add to the risk stratification of patients with idiopathic pulmonary fibrosis and thus improve clinical outcomes.

Whole blood and peripheral blood mononuclear cells represent the best sources for discovering a minimally invasive biomarker.^14^ Almost every immune cell type has been suggested to play an important role in the pathogenesis of idiopathic pulmonary fibrosis, including neutrophils,^15^ monocytes,^20^ regulatory T cells,^21^, ^22^ and B cells.^23^ However, multiple clinical trials have shown that immunosuppressants such as steroids do not slow disease progression,^24^ and might even lead to reduced survival.^24^, ^25^ In this cohort study, we aimed to investigate whether a specific immune cell type from peripheral blood mononuclear cell (PBMC) samples from patients with idiopathic pulmonary fibrosis could identify those at higher risk of poor outcomes. We then sought to validate our findings using cellular phenotyping by flow cytometry, cytometry by time of flight, and electronic health records.

## Methods

### Study design and discovery cohort

**Figure 1:**
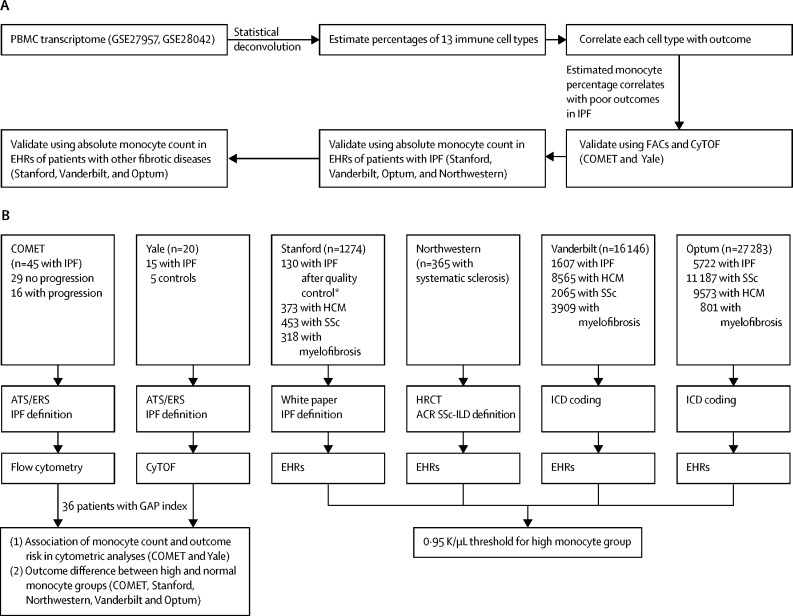
Analysis overview Overview of discovery and validation analyses (A) and detailed information for each validation cohort including the number of samples, diagnostic criteria for idiopathic pulmonary fibrosis (IPF), and data modality used for measuring monocytes (B). EHR=electronic health records. PBMC=peripheral blood mononuclear cell. FACs=fluorescence-activated cell sorting. CyTOF=cytometry by time-of-flight. ATS and ERS=American Thoracic Society and European Respiratory Society. HRCT=high-resolution CT. ACR=American College of Rheumatology. SSc-ILD=scleroderma-associated interstitial lung disease. HCM=hypertrophic cardiomyopathy. ICD=International Classification of Diseases. GAP=gender, age, physiology. *Of the 153 patients in the Stanford cohort initially identified with ICD codes as having idiopathic pulmonary fibrosis, 151 had charts available, of whom 130 had confirmed idiopathic pulmonary fibrosis. We removed data for the remaining 21 patients from all analyses.

Figure 1 provides an overview of our analyses. First, we did a discovery analysis with statistical devolution of publicly available whole transcriptome microarray data in the Gene Expression Omnibus at the National Center for Biotechnology Information (NCBI GEO)^26^ from the PBMC samples of 120 patients with idiopathic pulmonary fibrosis^9^, ^27^ to estimate the percentages of 13 immune cell types in each patient.

Transcriptome data were from the University of Pittsburg (PA, USA; NCBI GEO identifier GSE28042; n=75) and the University of Chicago (IL, USA; GSE27957; n=45). These cohorts have been previously described.^15^ Briefly, diagnosis of idiopathic pulmonary fibrosis in both cohorts was established by a multidisciplinary group at each institution with the American Thoracic Society/European Respiratory Society criteria. Patients were followed up in clinics (at 3-month to 4-month intervals) from blood draw until death or completion of the study on Feb 5, 2011.

We used immunoStates as a basis matrix with support vector regression to estimate percentages of each immune cell type in the PBMC sample at diagnosis. Next, we did a survival analysis to identify which types of immune cell were associated with poor outcomes in patients with idiopathic pulmonary fibrosis, using transplant-free survival with transplantations and deaths as events (figure 1). This analysis identified high proportion of monocytes in a sample as a potential prognostic biomarker of poor outcomes in idiopathic pulmonary fibrosis.

### Validation cohorts

We validated the association between poor outcomes in patients with idiopathic pulmonary fibrosis and high monocyte counts (assessed with flow cytometry or cytometry by time of flight) in two independent cohorts (COMET trial [appendix]^18^, ^23^, ^28^ and Yale). We further assessed the association between poor outcomes in patients with one of four fibrosis-related conditions and high monocyte count (assessed by absolute monocyte count as part of a full blood count) in three large health record databases and a cohort of patients with systemic sclerosis.

We used a cohort of patients from the COMET trial as a validation cohort to test whether increased monocyte count was associated with risk of idiopathic pulmonary fibrosis disease progression. The COMET trial profiled peripheral blood mononuclear cell samples from 45 patients with idiopathic pulmonary fibrosis using flow cytometry from March 12, 2010, to March 10, 2011. Classical monocytes were identified by surface expression of CD14 and the absence of surface expression of CD16 (CD14+ CD16–). Patients were diagnosed as having idiopathic pulmonary fibrosis on the basis of characteristic CT scans or usual interstitial pneumonia pathology confirmed by lung biopsy. COMET excluded patients with idiopathic pulmonary fibrosis if they were older than 80 years, had been diagnosed with the disease for more than 4 years, had a collagen-vascular disorder, an FEV_1_/FVC ratio of less than 0·60, evidence of active infection at screening, or a high-risk comorbidity. Of the 45 patients, 36 had a GAP index calculated based on available data.^6^ 16 patients with idiopathic pulmonary fibrosis in the COMET trial had progressive disease, where progression was defined as any of the following: death, acute exacerbation of disease, lung transplantation, or relative decrease in FVC (L) of at least 10% or DL_CO_ (mL/min per mm Hg) of 15% by 48 weeks. The remaining 29 patients are referred to as having non-progressive disease.

We used the Yale cohort to test whether monocyte percentages were higher in high-risk patients with idiopathic pulmonary fibrosis, as defined by a previously described and prospectively validated 52-gene signature.^10^, ^24^ The Yale cohort profiled peripheral blood mononuclear cell samples from 15 patients with idiopathic pulmonary fibrosis and five healthy controls (appendix) using cytometry by time of flight, where monocytes were defined as CD14+ CD16– from April 28, 2014, to Aug 20, 2015. A multidisciplinary group diagnosed idiopathic pulmonary fibrosis with ATS/ERS criteria. Using the gene signature, six patients were defined as high risk and nine were defined as low risk.

Next, we analysed databases of electronic health records (EHRs) to investigate whether high absolute monocyte count obtained from complete blood counts is a prognostic marker of poor outcomes (assessed as all-cause mortality) in patients with idiopathic pulmonary fibrosis or other fibrotic diseases (systemic sclerosis, hypertrophic cardiomyopathy, and myelofibrosis; figure 1).

For this analysis, we used four databases: the Stanford Translational Research Integrated Database Environment^29^ (containing EHRs from 1·8 million adult and paediatric patients seen at Stanford University Medical Center from Jan 1, 2008, to Dec 31, 2015), the Vanderbilt University Medical Center Database^30^ (containing EHRs from 2·8 million adult and paediatric patients seen at Vanderbilt University Medical Center from Jan 1, 2008, to Dec 31, 2016), the Optum Clinformatics DataMart^31^ (a national insurance claims database of 63 million US residents from Jan 1, 2004, to Dec 31, 2016), and the Northwestern SSc-ILD cohort (365 patients with systemic sclerosis and confirmed interstitial lung disease as defined by clinical assessment, pulmonary function, and high-resolution chest CT from Feb 15, 2001 to July 31, 2017). We created a cohort for each of the four fibrotic disorders in the Stanford, Vanderbilt, and Optum EHR databases using International Classification of Diseases (ICD) codes to identify each disease (appendix) and included all Northwestern patients as systemic sclerosis. Patients were required to have an absolute monocyte count as part of a complete blood count within 30 days before or after diagnosis in the Stanford, Vanderbilt, and Optum cohorts. No date of diagnosis was available for the Northwestern cohort. Therefore, the absolute monocyte count closest to the first recorded pulmonary function test was used. We used a cutoff of 0·95 K/μL or greater to indicate high monocyte count.

To ensure quality control for the correct identification of patients with idiopathic pulmonary fibrosis by ICD-9 coding, we obtained patient charts for the Stanford cohort to calculate the positive predictive value of the ICD coding for a diagnosis of idiopathic pulmonary fibrosis. We considered a confirmed diagnosis as the presence of a radiology report stating probable or typical usual interstitial pneumonia on CT, alongside a clinical note confirming the diagnosis of idiopathic pulmonary fibrosis as recommended by the latest diagnostic criteria.^32^ We examined the positive predictive value of idiopathic pulmonary fibrosis ICD-9 code 516.31 in the Stanford cohort to estimate accuracy of ICD codes for patient identification.

Finally, we explored whether high absolute innate immune cell counts were specific for idiopathic pulmonary fibrosis or general markers of mortality by creating propensity-matched control cohorts without idiopathic pulmonary fibrosis from the Stanford and Optum databases. Control cohorts were matched to idiopathic pulmonary fibrosis cohorts by age, sex, number of diagnoses, number of visits, and duration of observation (appendix).

Studies were approved by institutional review boards at each institution and written informed consent was obtained from all patients. We received institutional approval for the review of patient charts at Stanford (IRB-45187).

### Sample collection and measurements

Samples were collected and processed with flow cytometry in the COMET cohort from March 12, 2010, to March 10, 2011, as previously described^21^ (appendix). To our knowledge, the current study is the first report of the absolute monocyte counts from the COMET cohort. Peripheral blood mononuclear cell samples from patients with idiopathic pulmonary fibrosis in the Yale cohort were collected as previously described.^19^ We measured CD14+ CD16– classical monocyte expression by cytometry by time of flight analysis (appendix). We obtained monocyte counts in EHR cohorts from complete blood counts in patient records. We used the 742-7 code from the Logical Observation Identifier Names and Codes for identifying monocyte counts in EHR records.

### Outcomes

The primary outcome for the discovery cohort was transplant-free survival. As in previous analyses of the COMET cohort, the primary outcome for this cohort was progression of idiopathic pulmonary fibrosis, defined as any of the following: death, acute exacerbation of idiopathic pulmonary fibrosis, lung transplantation, or relative decrease in FVC of at least 10% or DL_CO_ of 15% by 48 weeks. The Yale cohort did not have outcome data; instead, patients were classified as high risk (n=6) and low risk (n=9) using a previously described and prospectively validated 52-gene signature.^9^, ^19^ All-cause mortality was the primary outcome in the EHR cohorts of Stanford, Vanderbilt, Optum, and Northwestern.

### Statistical analysis

We used statistical deconvolution of peripheral blood mononuclear cell transcriptome data to estimate the proportions of 13 immune cell types.^27^, ^28^ Statistical deconvolution estimates the percentage of various cell types in a whole transcriptome profile obtained from a mixture of various cell types (eg, a peripheral blood mononuclear cell sample includes immune cells such as monocytes, T cells, B cells, and natural killer cells). It uses a set of pre-defined genes that represent cell types of interest, known as a basis matrix, and typically a variant of regression. Here, we used immunoStates as a basis matrix because it has been shown to reduce the effect of the biological and technical heterogeneity in transcriptome data on statistical deconvolution and identify robust changes in immune cell proportions.^27^, ^28^, ^33^ We used support vector regression with immunoStates for statistical deconvolution of the transcriptome from peripheral blood mononuclear cell samples from patients with idiopathic pulmonary fibrosis.

Before deconvolution, we modified immunoStates to remove seven cell types that were highly unlikely to be present in a peripheral blood mononuclear cell sample including neutrophils, basophils, eosinophils, mast cells, and macrophages. After estimating proportions of the 13 immune cell types, we grouped the cell subtypes into major immune cell types that were present in a sample as follows: (1) CD4+, CD8+, and γ-δ T cells were grouped as T cells, (2) naive B cells, memory B cells, and plasma cells as B cells, and (3) CD14+ cells as classical monocytes.

For each of the three major immune cell types (monocytes, T cells, and B cells) estimated using deconvolution, we stratified the patients with idiopathic pulmonary fibrosis into two groups based on their estimated cell percentages relative to the mean. We could not use absolute counts because statistical deconvolution only estimates percentages. We used transplant-free survival as the outcome, with both transplantation and death considered as events. We used the Cox proportional hazard model to determine hazard ratios (HRs). All analyses were done with R (version 3.3.1) statistical programming language with the packages survival (2.41.3) and rmeta (3.0).

All analyses of the COMET cohort used the first recorded CD14+ monocyte count for each patient. We used two-sided student's t test to compare monocyte counts between patient groups in both COMET and Yale. In EHR cohorts, we used all-cause mortality as the outcome. In clinical practice, normal monocyte count ranges between 0·2 K/μL and 0·95 K/μL.^34^ Therefore, in each EHR cohort, we grouped patients with absolute monocyte counts greater than 0·95 K/μL in the high monocyte group, and the rest in the normal monocyte group. We used absolute cell count instead of proportion because absolute counts of each cell type can be considered independently from one another. The variables used in the Cox regression were selected based on available data. We found that none of the covariates used in our analysis (age, sex, GAP index, FVC, or monocyte count) violated proportional hazards assumption of a Cox regression.^35^ All analysis was done in the R statistical programming language. We used the cox.zph function in the R package survival (version 2.41.2) for testing proportional hazards assumption of a Cox regression.

We analysed the sensitivity of HR by varying the threshold for high monocyte count in each EHR cohort (Stanford, Vanderbilt, Optum) for each fibrotic disease in increments of 0·05 K/μL. We required that one of the two patient groups (normal monocyte count *vs* high monocyte count) contained at least 3% of the corresponding cohort. We used DerSimonian-Laird^36^ to estimate heterogeneity in the HRs between cohorts and used a random effects inverse variance model for calculating the summary HR. All analyses were done with R, version 3.3.1.

### Role of the funding source

The funders of the study had no role in study design, data collection, data analysis, data interpretation or writing of the report. MKDS and PK had full access to all the data in the study and had final responsibility for the decision to submit for publication.

## Results

**Figure 2:**
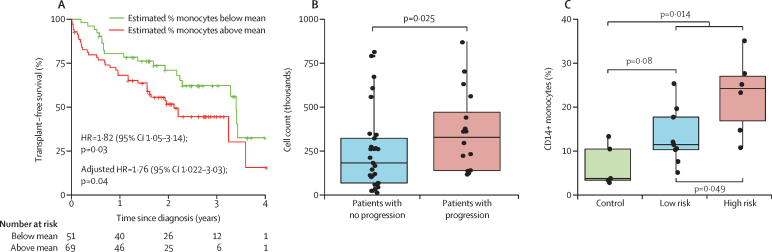
Classic monocyte (CD14+ CD16–) count association with poor outcomes in patients with idiopathic pulmonary fibrosis (A) In the discovery analysis, patients with an estimated proportion of classical monocytes greater than the mean had reduced transplant-free survival. (B) Patients with progressive idiopathic pulmonary fibrosis in the COMET trial had higher CD14+ monocyte counts than patients with non-progressive disease. (C) Patients with idiopathic pulmonary fibrosis in the Yale cohort had higher proportions of CD14+ monocytes (of total peripheral blood mononuclear cells) compared with healthy controls. Each circle represents an individual patient. Horizontal lines within boxes indicate the median. The bottom and top edges of each box indicate the first and third quartiles of the data, respectively. Vertical lines indicate the datapoints within 1·5 times of the IQR of the data.

We began our discovery analysis with deconvolution of publicly available transcriptome data in the NCBI GEO database from 120 samples of PBMCs from patients with idiopathic pulmonary fibrosis. Estimated CD14+ classical monocyte percentages above the mean were significantly associated with shorter transplant-free survival (HR 1·82, 95% CI [1·05–3·14]; figure 2), whereas there was no association with estimated T cell and B cell percentages (0·97, 0·59–1·66 and 0·78, 0·45–1·34, respectively; appendix).

In our validation analysis of flow cytometry data for 45 patients with idiopathic pulmonary fibrosis from the COMET trial, absolute count of CD14+ monocytes was significantly higher in patients with progressive disease compared with those with non-progressive disease (p=0·025, figure 2). Patients with idiopathic pulmonary fibrosis in the Yale cohort had higher proportions of CD14+ monocytes (of PBMCs) than healthy controls (p=0·014, figure 2), with percentage of CD14+ cells increasing as mortality risk increased (n=20). Additionally, the six high-risk patients with idiopathic pulmonary fibrosis had more CD14+ monocytes than the nine low-risk patients (p=0·049, figure 2).

Of 153 patients in the Stanford cohort, 151 had charts available to examine. We confirmed the diagnosis of idiopathic pulmonary fibrosis in 130 patients (positive predictive value [PPV] 86%) by a radiology report stating probable or typical usual interstitial pneumonia on CT alongside a clinical note confirming the diagnosis of idiopathic pulmonary fibrosis as recommended by the latest diagnostic criteria for idiopathic pulmonary fibrosis.^31^ Thus, there were 130, 1607, and 5722 patients with idiopathic pulmonary fibrosis in the Stanford, Vanderbilt and Optum cohorts, respectively, who matched the inclusion criteria (appendix). The absolute cutoff of 0·95 K/μL for high monocyte count corresponded to the 90th, 82nd, and 88th percentiles in the Stanford, Vanderbilt, and Optum cohorts for patients included in this study, respectively, at the time of diagnosis (appendix).

**Figure 3:**
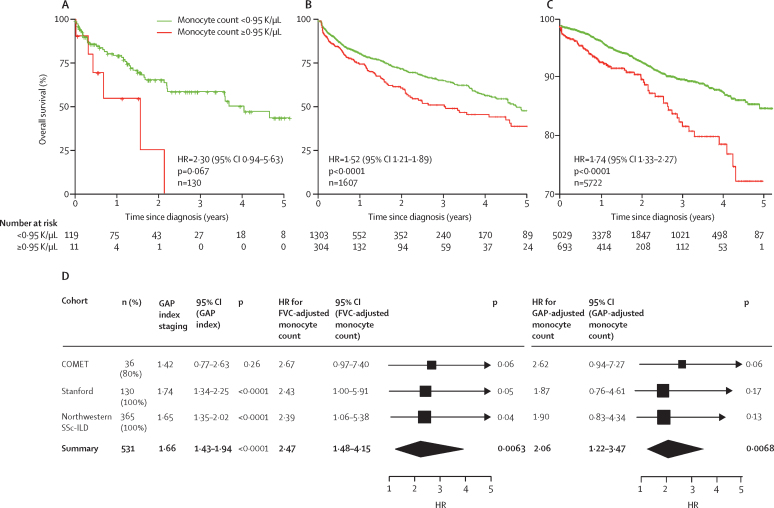
Survival of patients with idiopathic pulmonary fibrosis patients up to 5 years after diagnosis Data are stratified by absolute monocyte count ≥0·95 K/μL or <0·95 K/μL. With lung transplantation as a censoring event and after adjusting for age and sex, monocyte count ≥0·95 K/μL was significantly associated with increased risk of mortality in the Stanford (A), Vanderbilt (B), and Optum cohorts (C). Hazard ratios (HRs) for monocyte counts and outcomes across the COMET, Stanford, and Northwestern systemic sclerosis with interstitial lung disease (SSc-ILD) cohorts, adjusted for forced vital capacity (FVC) and gender, age, and physiology (GAP) index (D). Bold text shows the combined HRs for poor outcomes across the three cohorts. There was no heterogeneity in FVC-adjusted and GAP-adjusted HRs across the three cohorts.

With lung transplantation as a censoring event, after adjusting for age at diagnosis and sex, high monocyte count was associated with increased risk of all-cause mortality in patients with idiopathic pulmonary fibrosis (figure 3, table). HRs for high absolute monocyte count in each of the three EHR cohorts were not affected by steroid use (data not shown). Increased absolute neutrophil count also showed an association with survival (Stanford HR 2·55, 95% CI 1·1–5·8, p=0·026; Optum 2·83, 2·2–3·6, p<0·0001; appendix). Additionally, absolute lymphocyte count was not significantly associated with survival in any of the three cohorts (appendix).

**Table:** Hazard ratios in study cohorts with idiopathic pulmonary fibrosis for GAP index and monocytes

	**Hazard ratio**	**95% CI**	**p value**
**COMET (n=36)**			
GAP index	1·42	0·77–2·63	0·26
Monocytes	2·65	0·95–7·34	0·060
Monocytes (age and sex corrected)	2·46	0·86–6·98	0·092
Monocytes (FVC-corrected)	2·69	0·97–7·45	0·057
Monocytes (GAP-corrected)	2·64	0·95–7·33	0·062
**Stanford (n=130)**			
GAP index	1·73	1·34–2·25	<0·0001
Monocytes	2·83	1·17–6·81	0·026
Monocytes (age and sex corrected)	2·30	0·94–5·63	0·067
Monocytes (FVC-corrected)	2·43	1·00–5·91	0·049
Monocytes (GAP-corrected)	1·87	0·76–4·61	0·17
**Vanderbilt (n=1607)**			
Monocytes	1·46	1·17–1·82	0·00094
Monocytes (age and sex corrected)	1·52	1·21–1·89	<0·0001
**Optum (n=5722)**			
Monocytes	1·96	1·50–2·56	<0·0001
Monocytes (age and sex corrected)	1·74	1·33–2·27	<0·0001
**Northwestern (n=365)**			
GAP index	1·65	1·35–2·02	<0·0001
Monocytes	2·25	1·01–5·04	0·049
Monocytes (age and sex corrected)	2·06	0·91–4·69	0·082
Monocytes (FVC-corrected)	2·39	1·06–5·38	0·035
Monocytes (GAP-corrected)	1·90	0·83–4·36	0·13

Data are shown with and without correction for age, sex, predicted forced vital capacity (FVC), and GAP (gender, age, and physiology index).

We investigated whether monocyte count was a risk factor for mortality independent of idiopathic pulmonary fibrosis severity measured as FVC or GAP. For this purpose, we used three cohorts (Stanford, Northwestern, and COMET) that contained patients with confirmed diagnosis of idiopathic pulmonary fibrosis, unlike the Vanderbilt and Optum cohorts for which we used ICD codes to identify patients with idiopathic pulmonary fibrosis. These three cohorts contained 36 of the 45 patients with idiopathic pulmonary fibrosis in the COMET trial with baseline pulmonary function tests, 130 patients with idiopathic pulmonary fibrosis from Stanford University, and 365 patients with systemic sclerosis with interstitial lung disease confirmed by pulmonary function tests and chest CT from Northwestern University (appendix). Overall baseline GAP index was associated with poor outcomes, although this was not statistically significant in the COMET trial (figure 3). In each of the three cohorts, HR for GAP index was less than 1·75. By contrast, after adjusting for FVC, monocyte count of 0·95 K/μL or greater was associated with poor outcomes in pulmonary fibrosis in the COMET trial, the Stanford cohort, and the Northwestern cohort. When combining FVC-adjusted HRs across the three cohorts, high monocyte counts were significantly associated with poor outcomes in patients with idiopathic pulmonary fibrosis (figure 3).

When adjusting for GAP index, monocyte count was not significantly associated with poor outcomes in individual cohorts (figure 3). However, when combining GAP index-adjusted HRs across the three cohorts, high monocyte count was significantly associated with poor outcomes in patients with pulmonary fibrosis (figure 3). There was no heterogeneity in HRs across the three cohorts when combining using random effects inverse variance meta-analysis (FVC-adjusted HR Cochran's Q=0·2, p=0·9; GAP index-adjusted HR Cochran's Q=1·46, p=0·48).

Using 0·95 K/μL as a threshold for high monocyte count in matched non-idiopathic pulmonary fibrosis cohorts, absolute monocyte count was not associated with an increased risk of mortality (Stanford HR 1·18, 95% CI 0·64–2·16; Optum 0·91, 0·67–1·23; appendix). By contrast, neutrophil count continued to predict mortality in matched non-fibrotic cohorts (appendix). Collectively, these results suggest that high absolute monocyte count is an idiopathic pulmonary fibrosis-specific marker of mortality and poor outcomes, whereas high neutrophil count is a general marker of mortality.

There was no correlation between change in monocyte count over time and survival by repeated measures ANOVA (data not shown). Instead, monocyte counts for individual patients were stable over time, indicating that patients with idiopathic pulmonary fibrosis retained the same risk profile. Because a threshold value for high monocyte count is necessary in clinic to stratify patients into risk groups, we did a sensitivity analysis to confirm the robustness of the association to the threshold value (appendix). Risk of mortality increased with rising monocyte counts, suggesting that the relationship between monocyte count and survival risk is best viewed as a continuum (appendix).

Similar to the selection criteria for patients with idiopathic pulmonary fibrosis, the selection criteria for systemic sclerosis, hypertrophic cardiomyopathy, and myelofibrosis was a diagnosis of a fibrotic disease alongside a monocyte count within 30 days of diagnosis. There were 37 244 patients with one of the three fibrotic diseases in the three cohorts (Stanford 1144, Vanderbilt 14 539, and Optum 21 561) that met the criteria.

**Figure 4:**
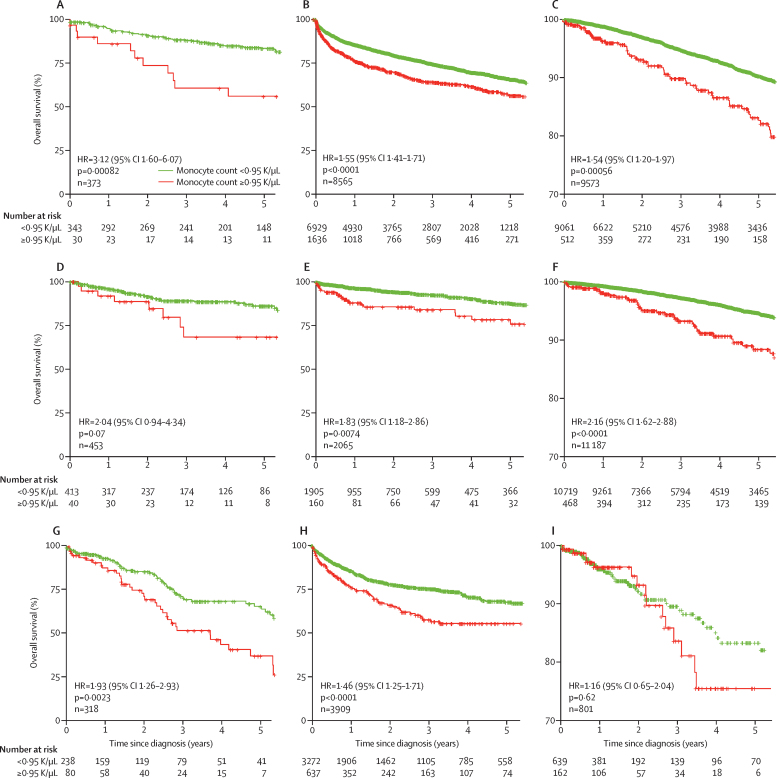
Association between high monocyte count in complete blood count at diagnosis with mortality in hypertrophic cardiomyopathy, systemic sclerosis, and myelofibrosis Data are from electronic health records in the Stanford, Vanderbilt, and Optum cohorts. Absolute monocyte count ≥0·95 K/uL was significantly associated with reduced survival for patients with hypertrophic cardiomyopathy in Stanford (A), Vanderbilt (B), and Optum (C); systemic sclerosis in Stanford (D), Vanderbilt (E), and Optum (F); and myelofibrosis in Stanford (G), Vanderbilt (H), and Optum (I).

Higher absolute monocyte count was significantly associated with shortened survival in patients with hypertrophic cardiomyopathy across the Stanford, Vanderbilt, and Optum cohorts (figure 4). Higher absolute monocyte count was associated with shortened survival in systemic sclerosis cohorts from Vanderbilt and Optum (figure 4). In patients with myelofibrosis, higher absolute monocyte count was associated with shortened survival in Stanford and Vanderbilt but not in Optum (figure 4). Further analysis showed that the Optum cohort of patients with myelofibrosis had a much higher monocyte count than any of the other cohorts (data not shown). Additionally, the number of patients with myelofibrosis in the Vanderbilt cohort was substantially larger than expected based on national prevalence of myelofibrosis of four to six per 100 000.^37^ We re-defined the high monocyte group as those with the highest 10% of absolute monocyte count within the Optum cohort of patients with myelofibrosis (1·4 K/μL), and the Stanford cohort (1·6 K/μL). In both cohorts, the high monocyte group had an increased risk of mortality (Stanford: HR 2·6, 95% CI 1·5–4·5, p=0.00051; Optum 1·94, 1·0–3·7, p=0·039; appendix). Collectively, these results provide evidence that a high absolute monocyte count within 30 days of a diagnosis of a fibrotic disease could be prognostic of shortened patient survival across fibrotic diseases.

## Discussion

We identified classical monocytes as a prognostic marker in patients with idiopathic pulmonary fibrosis. Increased monocyte count at the time of diagnosis continued to be associated with poor outcomes in pulmonary fibrosis after adjusting for FVC and GAP, and was also associated with increased mortality in non-pulmonary fibrotic diseases. Most importantly, we showed that a single threshold value of absolute monocyte counts of 0·95 K/μL could be used to identify high-risk patients with a fibrotic disease. Our study addresses a clinical need for a prognostic biomarker for idiopathic pulmonary fibrosis, which would enable more conscientious allocation of scarce resources, including lung transplantations and at-risk patient monitoring. Although there has been much effort to identify prognostic biomarkers for individual fibrotic diseases, many of the published biomarkers are gene panels and multicytokine signatures that are expensive and labour intensive.^10^ By contrast, absolute monocyte count is routinely measured as part of a complete blood count, an inexpensive test used in clinical practice worldwide. Finally, the findings from our study suggest a strategy to combine existing molecular data with EHR data to improve clinical practice.

Monocytes are known to contribute to the pathogenesis of idiopathic pulmonary fibrosis.^38^, ^39^ The robust association of high monocyte count with mortality in other fibrotic diseases such as systemic sclerosis, hypertrophic cardiomyopathy, and myelofibrosis suggests they might contribute to pathogenesis of these diseases as well. Recruitment of monocytes to damaged tissue to aid in repair—typically a beneficial response—becomes detrimental when an organ undergoes continuous, pathogenic wound healing.^40^ In idiopathic pulmonary fibrosis, monocytes migrate to the lung and become profibrotic inflammatory alveolar macrophages.^39^ Consistent with this model of lung fibrosis, depletion of monocytes has been shown to strikingly reduce the degree of fibrosis following lung injury in the bleomycin treatment-induced lung fibrosis mouse model.^39^ There are no clinically approved drugs that reduce monocyte count without causing neutropenia, and depletion of monocytes could perhaps lead to increased risk of infection.^39^ However, patients with idiopathic pulmonary fibrosis with monocyte counts in the normal range have significantly longer survival, suggesting that reducing monocyte counts of patients to normal range could increase survival without increasing the risk of infection. Hence, selective depletion of monocytes might represent a new avenue of therapy in patients with idiopathic pulmonary fibrosis.

Patients with a high monocyte count at diagnosis maintained their high count through the course of their disease (data not shown). This observation is supported by previous reports^19^ that patients with idiopathic pulmonary fibrosis who have a high-risk peripheral blood gene expression signature continued to display the same risk profile when retested. By contrast, fibrotic disease severity indices such as pulmonary function tests in idiopathic pulmonary fibrosis, ejection fraction rate in hypertrophic cardiomyopathy, or the modified Rodnan skin score in systemic sclerosis worsen in patients over time. This observation suggests that monocyte count might identify a patient with a fibrotic disease who is at high risk of mortality earlier than fibrotic disease-severity indices. A systematic comparison of such indices and monocyte counts at early disease stages is required to further support this observation.

Monocyte count was not predictive of mortality in our propensity matched cohorts of patients without idiopathic pulmonary fibrosis. This result aligns with previous findings^41^, ^42^, ^43^ showing that monocyte count is not a mortality risk factor in heart disease or stroke, the main cause of death in the USA. Unlike monocytes, neutrophil count predicted death in fibrotic diseases and their matched cohorts, suggesting that neutrophil count is a general marker of death, not specific to fibrotic disorders. Furthermore, neutrophil depletion has been shown to exacerbate the progression of fibrosis in vivo,^44^, ^45^ suggesting that neutrophils might not play a mechanistic role in progression of lung fibrosis.

Our study had some limitations. First, two of our six validation cohorts (Vanderbilt and Optum) used only ICD codes for identifying patients with a fibrotic disease. Therefore, we might have missed some patients and inappropriately included others. In the Stanford idiopathic pulmonary fibrosis cohort, which also used ICD codes to identify patients, individual chart review confirmed 130 out of 153 patients had idiopathic pulmonary fibrosis, with a PPV of 86%. This PPV is excellent for a rare disease.^46^ Overall, our study included four validation cohorts totalling 555 patients with confirmed pulmonary fibrosis (idiopathic pulmonary fibrosis and Northwestern systemic sclerosis-associated interstitial lung disease cohorts) to increase the accuracy. HRs for each of the fibrotic diseases, when using ICD codes, were 1·5 or more in all but one cohort. By contrast, in three idiopathic pulmonary fibrosis cohorts with confirmed diagnosis (COMET, Stanford, and Northwestern), HRs were at least 2·39 after adjusting for FVC and at least 1·87 after adjusting for GAP. These results suggest that inclusion of incorrectly diagnosed patients using ICD codes might reduce the signal to bias HR towards null hypothesis, but not inflate it; and that despite the errors introduced by ICD codes, the signal-to-noise ratio in Vanderbilt and Optum cohorts was strong enough for monocyte count to be consistently observed as a risk factor. Second, we did not have information about disease severity or outcome for three of the six validation cohorts (Yale, Vanderbilt, and Optum cohorts). However, in the remaining three idiopathic pulmonary fibrosis cohorts with pulmonary function information, monocyte count was predictive of poor outcomes independent of disease severity. Third, our analysis was limited to idiopathic, high-morbidity fibrotic diseases and excluded other causes of fibrosis with a known pathogenesis such as radiation or alcoholic cirrhosis. The natural extension of our study would be to investigate association between high monocyte count and reduced mortality across all causes of organ fibrosis. Fourth, typically in idiopathic pulmonary fibrosis cohorts, marked predominance of men is reported. Although the COMET, Yale, and Stanford idiopathic pulmonary fibrosis cohorts comprised of more than 62% men, the Vanderbilt and Optum cohorts had substantially fewer men. It is possible that the proportion of men was lower in these cohorts because of patients incorrectly included due to the use of ICD codes.

Most importantly, our approach of estimating cellular proportions from publicly available transcriptome data and validating with complete blood counts from nation-wide EHRs and claims data presents a new strategy for leveraging existing molecular and clinical data to improve clinical practice. Large public data repositories such as the NCBI GEO^26^ include transcriptome data for thousands of whole blood and PBMC samples from many diseases. Statistical deconvolution of these data could identify important changes in immune cells, which in turn could be correlated with various disease outcomes using data from EHRs and claims to readily increase the prognostic usefulness of routinely performed laboratory tests.
