# Supplementary material for: Increased monocyte count as a cellular biomarker for poor outcomes in fibrotic diseases: a retrospective, multicentre cohort study
Source: Lancet Respir Med. 2019 Jun;7(6):497–508. doi: 10.1016/S2213-2600(18)30508-3 (PMC6529612; doi:10.1016/S2213-2600(18)30508-3)
Supplement: Supplementary appendix [file mmc1.pdf]

THE LANCET
Respiratory Medicine

Supplementary appendix

This appendix formed part of the original submission and has been peer reviewed. We post it as supplied by the authors.

Supplement to: Scott MKD, Quinn K, Li Q, et al. Increased monocyte count as a cellular biomarker for poor outcomes in fibrotic diseases: a retrospective, multicentre cohort study. *Lancet Respir Med* 2019; published online March 29. http://dx.doi.org/10.1016/S2213-2600(18)30508-3.

Appendix
Supplemental Methods

Validation cohorts

COMET trial

The COMET trial is a multi-center, prospective observational cohort study of IPF patients followed prospectively at 16-week intervals up to 80 weeks (http://www.clinicaltrials.gov, clinical trial ID no. NCT01071707). Patients were diagnosed as having IPF on the basis of characteristic CT scans or UIP pathology confirmed by lung biopsy. All subjects underwent baseline lung physiology testing (DLco, FVC, and 6-minute walk testing) and high-resolution computed tomography assessment. Patients were allowed to remain on current treatments. For this analysis, progression was defined any of the following: death, acute exacerbation of IPF, lung transplantation, or relative change in forced vital capacity (FVC, liters) of ≥10% or DL_{CO} (ml min^{-1} $mmHg^{-1}$) of 15% by 48 weeks. All sites received local Institutional Review Board approval. Samples were stained with CD14, CD16 and other markers as described previously.

The COMET trial profiled PBMC samples from 45 patients with IPF using flow cytometry, of which 16 met the criteria for progression ("progressors") and 29 did not ("non-progressors"). Although progressor/non-progressor status for each of the 45 patients with IPF was available, either FVC or DLCO values were not available for 9 patients, which were excluded from every survival analysis.

Yale Cohort

PBMC samples from 15 IPF patients and 5 healthy controls were barcoded with unique palladium-based combinatorial labels and stained as a single multiplexed sample. An antibody panel composed of 30 protein parameters (14 markers of immune cell phenotypes and 16 markers from a prospectively validated IPF-risk gene signature) were assayed simultaneously. Antibodies for classical monocytes, CD14-160Gd and CD16-165Ho, were simultaneously applied. Cell events were selected if cells had CD14 positive and CD16 negative expression.

EHR cohorts

We created a cohort for each of the four fibrotic disorders (IPF, SSc, HCM, and myelofibrosis) in each of the 3 EHR databases (Stanford, Vanderbilt, and Optum) as described below. We used the following ICD-9 and ICD-10 codes for IPF, SSc, HCM, and myelofibrosis in each of the 3 EHR databases:

1. IPF: ICD-9 – 516.31; ICD-10 – J84.112
2. SSc: ICD-9 – 710.1; ICD-10 – all child codes of M34 for SSc except M34.2 (SSc induced by drug and chemical)
3. HCM: ICD-9 – 425.1; ICD-10 – I42.1 or I42.2
4. Myelofibrosis: ICD-9 – 238.76; ICD-10 – D47.1
5. Lung transplant: ICD-9 – V42.6; ICD-10 – Z94.2

Patients matching these ICD codes were included if they had a complete blood count (CBC) within 30 days before or after the diagnosis. After identifying patients in each EHR database for each fibrotic disease, we discarded patients with absolute monocyte count < 0.05 K/uL. If a patient had multiple CBCs within 30 days of diagnosis, we only used the first CBC; for example, if an IPF patient had a CBC 25 days before and 7 days after the diagnosis of IPF, we used the CBC 25 days before the diagnosis. We stratified patients into "high monocyte" and "normal monocyte" groups by an absolute monocyte count threshold of 0.95 K/uL[43]. For IPF cohorts, we treated lung transplant as a censoring event. Any patient with a lab test after a reported death date was not included in the analysis.

Stanford EHR cohorts

The Stanford Translational Research Integrated Database Environment (STRIDE) contains EHRs from 1.8 million adult and pediatric patients seen at Stanford University Medical Center[42]. We analyzed EHRs from 2008-2015, and a total of 1297 patients from Stanford that met the initial inclusion criteria: 153 with IPF, 373 with HCM, 453 with SSc, and 318 with myelofibrosis. Clinical characteristics for the Stanford cohort are shown in **Supplementary Table 1**.

We used MatchIt R package to create 3:1 matched cohort to the Stanford IPF cohort based on age, sex, number of diagnoses, number of visits, and length of observation using the nearest neighbor non-parametric matching. **Supplementary Table 2** provides demographic information of propensity matched Stanford Cohort. **Supplementary Table 3** provides goodness of fit of propensity matched Stanford cohort.

Vanderbilt EHR cohorts

The Vanderbilt University Medical Center (VUMC) Database contains EHRs from 2.8 million adult and pediatric patients seen at Vanderbilt University Medical Center. We analyzed EHRs from 2008-2016. A total of 16,146 patients from Vanderbilt were included in the final analysis: 1,607 with IPF, 8,565 with HCM, 2,065 with SSc, and 3909 with myelofibrosis. Clinical characteristics for the Vanderbilt cohort are shown in **Supplementary Table 1**.

Optum EHR cohorts

The Optum Clinformatics™ data represents approximately 63 million US residents. We analyzed medical records from 2004-2016. A total of 27,283 patients were included in the Optum cohort: 5,722 with IPF, 11,187 with SSc, 9,573 with HCM, and 801 with myelofibrosis. Clinical characteristics for the Optum cohort are shown in **Supplementary Table 1**. However, the mortality information, which is linked from Social Security Administration death master files is incomplete, likely missing up to two-thirds of deaths[43]. To prevent the missing data from biasing our hazard ratios, we assumed that all patient cohorts had the same rate of death reporting. We used MatchIt R package to create 1:1 matched cohort to the Optum IPF cohort based on age, sex, number of diagnoses, number of visits, and length of observation using the nearest neighbor non-parametric matching. **Supplementary Table 2** provides demographic information of propensity matched Optum Cohort. **Supplementary Table 3** provides goodness of fit of propensity matched Optum cohort.

Northwestern SSc-ILD cohort

The Northwestern SSc-ILD cohort represents 365 patients that have confirmed systemic sclerosis with lung involvement as defined by clinical assessment, pulmonary function, and chest CT. We defined the date of diagnosis as the date of the first recorded pulmonary function test, and then chose the CBC test value closest to that date in order to most appropriately control for lung function in subsequent survival analyses. Length of observation was defined from the date of the first pulmonary function test to the last recorded interaction with the patient. Patient information was collected from 2001-2017.

Supplemental Figures

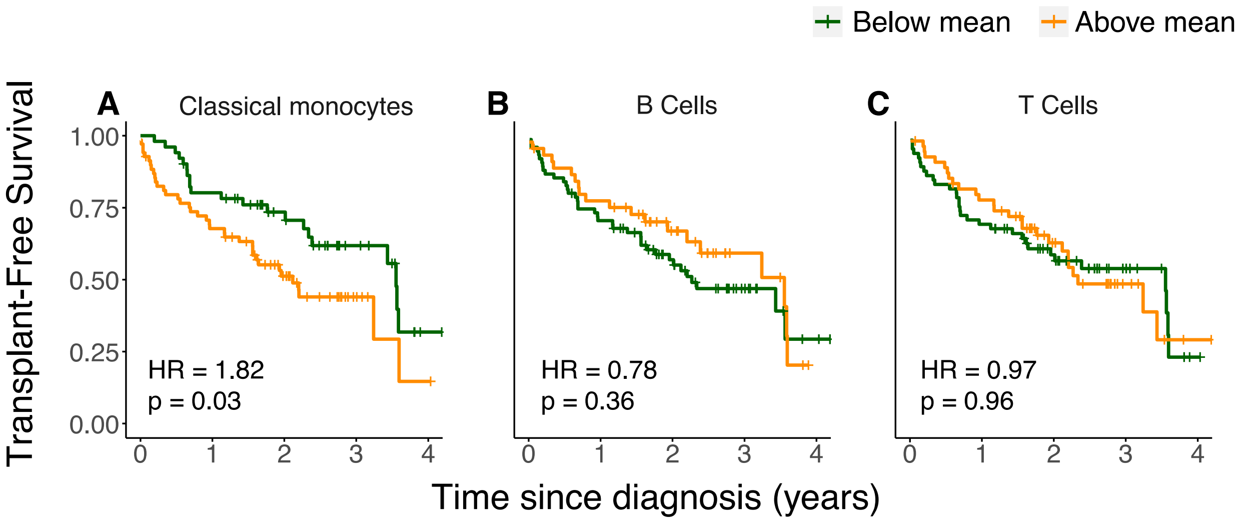

Supplementary Figure 1.

Survival of 120 IPF patients stratified by estimated PBMC leukocyte proportions. Overall survival was determined with patient with an estimated immune cell count above or below the mean for the cohort. A. Patients stratified by estimated classical (CD14+ CD16) monocytes. B. Patients stratified by estimated B-cell percentage. C. Patients stratified by estimated T-cell percentage.

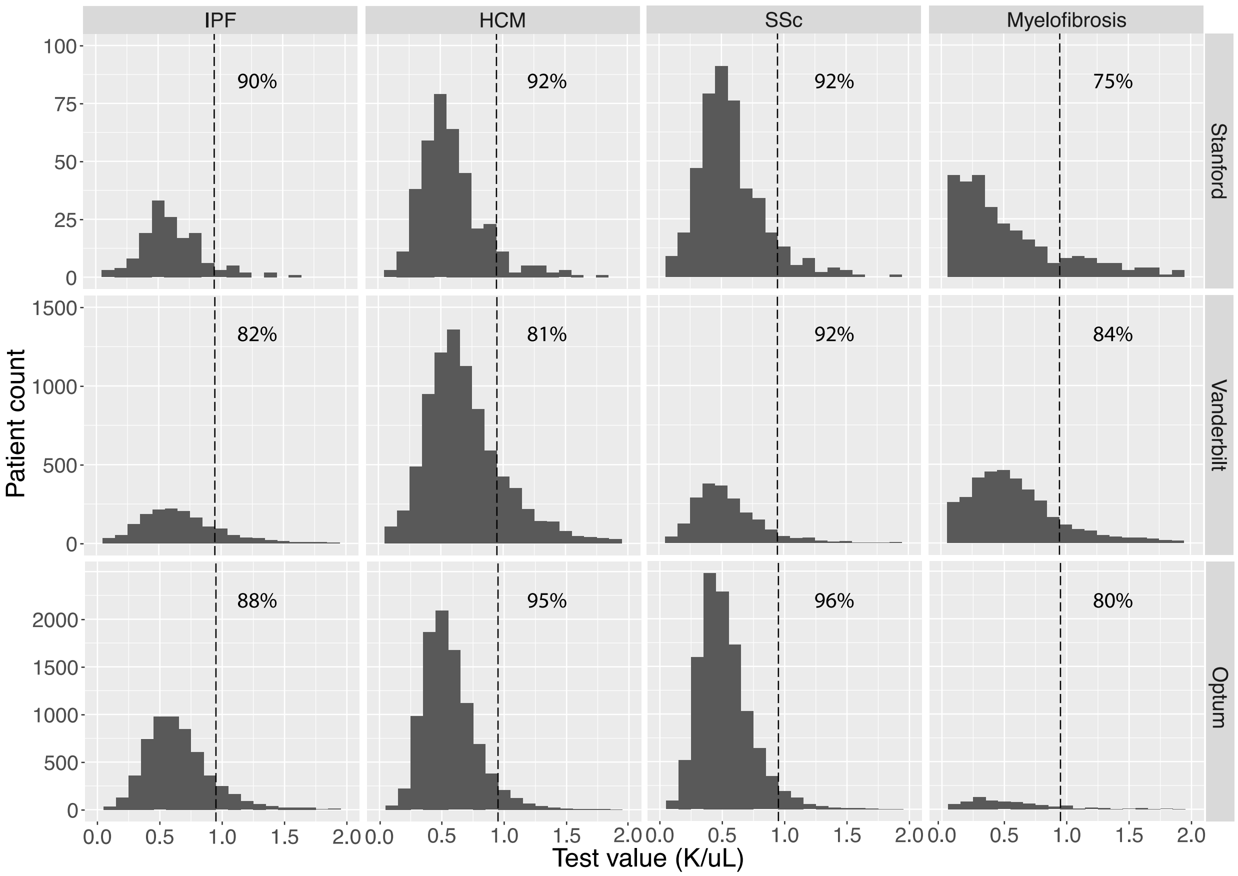

Supplementary Figure 2. Histogram of absolute monocyte counts for each disease and site cohort. The dotted line shows the 0.95 K/uL cutoff for a high monocyte count. Percentage of patients below the threshold are indicated by the text to the right of the dotted line. Each subplot shows the number of patients in the cohort, the median monocyte count, and the fraction of patients with monocyte counts below 0.95 K/uL.

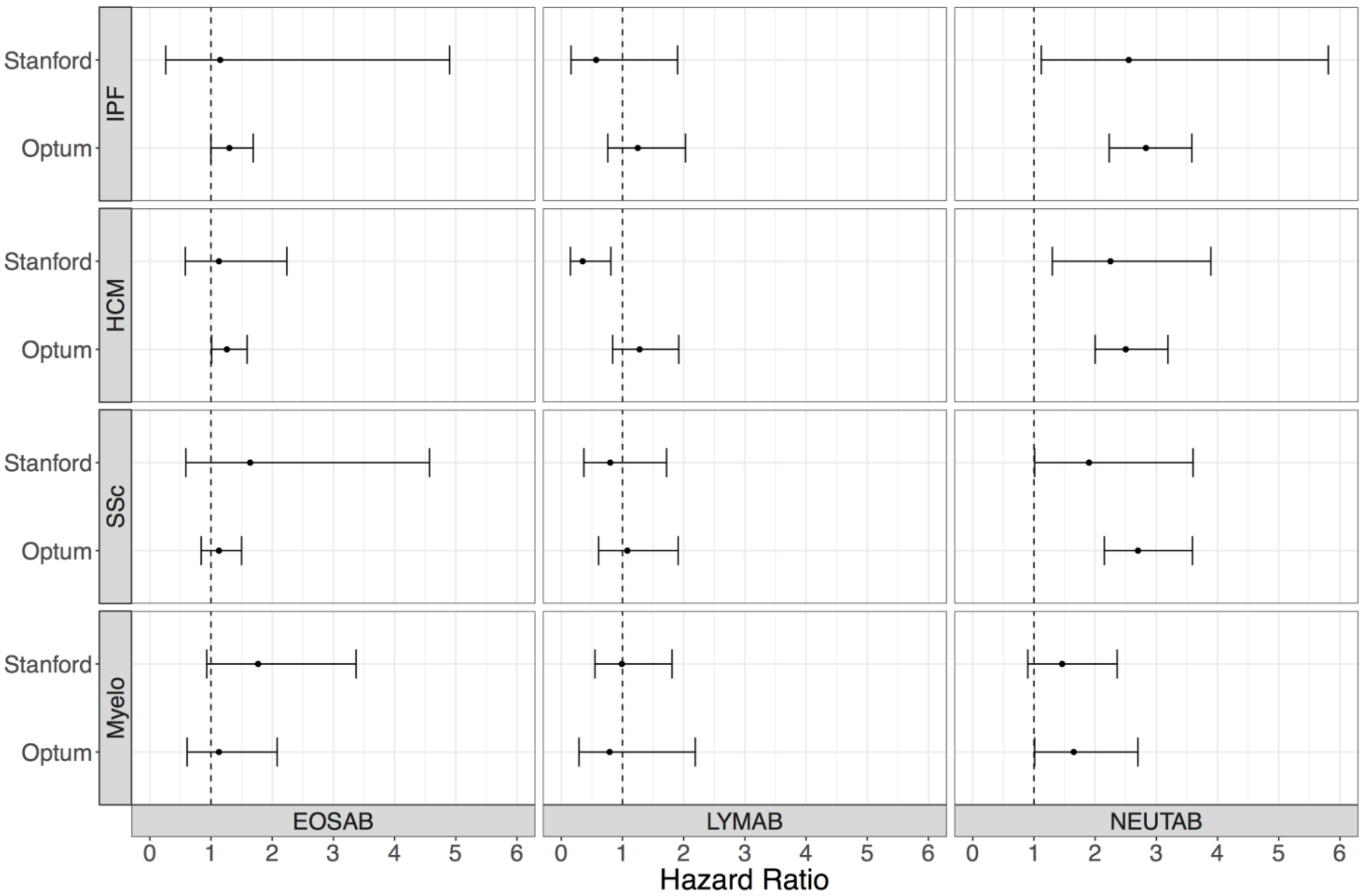

Supplementary Figure 3. Adjusted hazard ratio for survival for Stanford and Optum patients with absolute cell type counts (EOSAB = eosinophils, LYMAB = lymphocytes, NEUTAB = neutrophils) in the top decile, adjusted by age and sex across all diseases (IPF, HCM, SSc, and Myelofibrosis) in the Stanford and Optum cohorts.

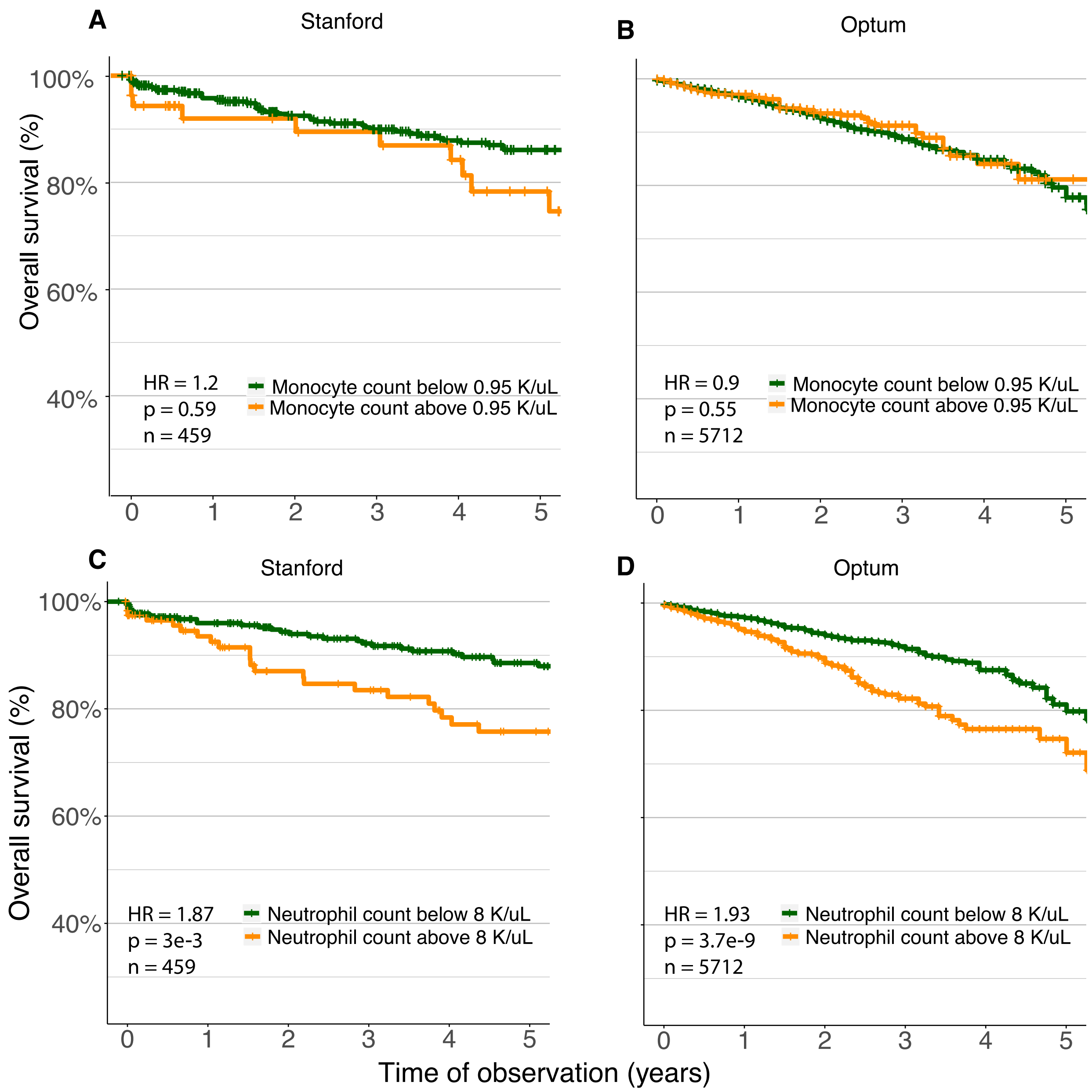

Supplementary Figure 4 A. Survival of propensity matched control cohorts stratified by absolute monocyte in Stanford (HR = 1.2 [95% CI: 0.6-2.0]) B. Optum (HR = 0.9 [95% CI: 0.7-1.2]). C. Survival of propensity matched control cohorts stratified by absolute neutrophil count in Stanford (HR = 1.87 [95% CI: 1.2-2.8]) D. Optum (HR = 1.93 [95% CI: 1.6-2.4]).

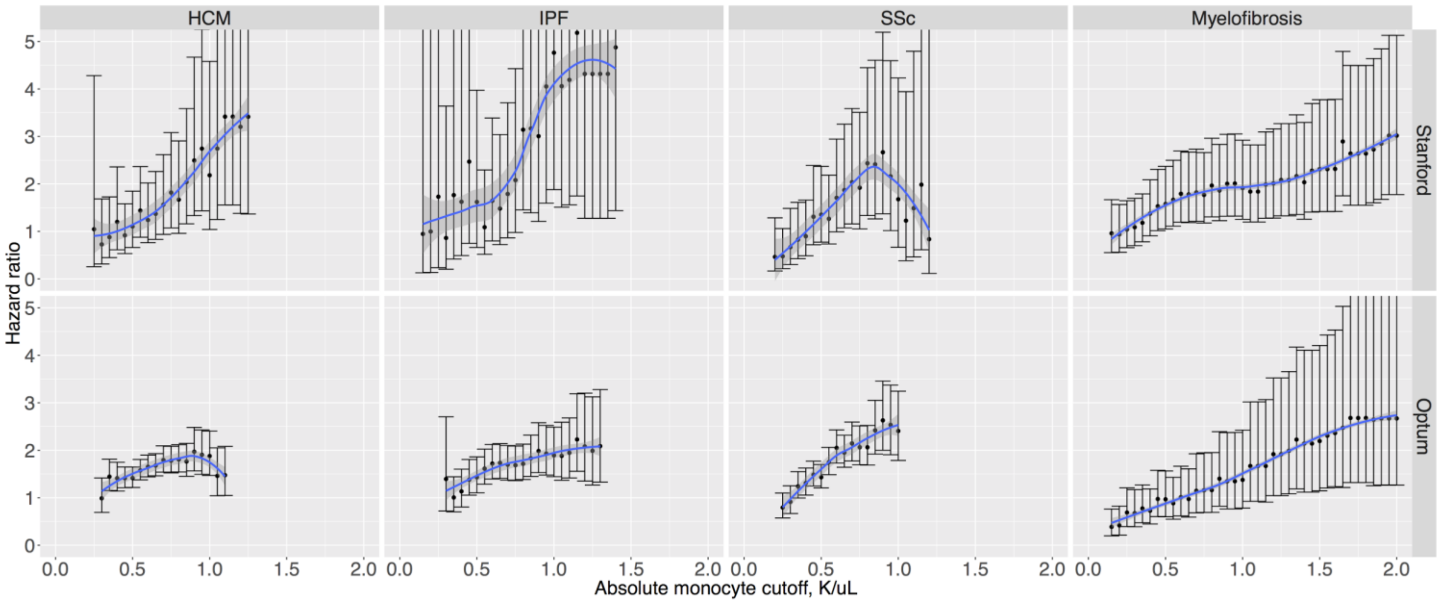

Supplementary Figure 5. Sensitivity of adjusted/raw hazard ratio to threshold defining "high" monocyte count in each disease (IPF, HCM, SSc, and Myelo) at Stanford and Optum. Error bars show the standard error. Hazard ratios tend to become significantly higher than 1.0 as the cutoff approaches or is above 0.95 K/uL. The association is robust to the particular selection of threshold.

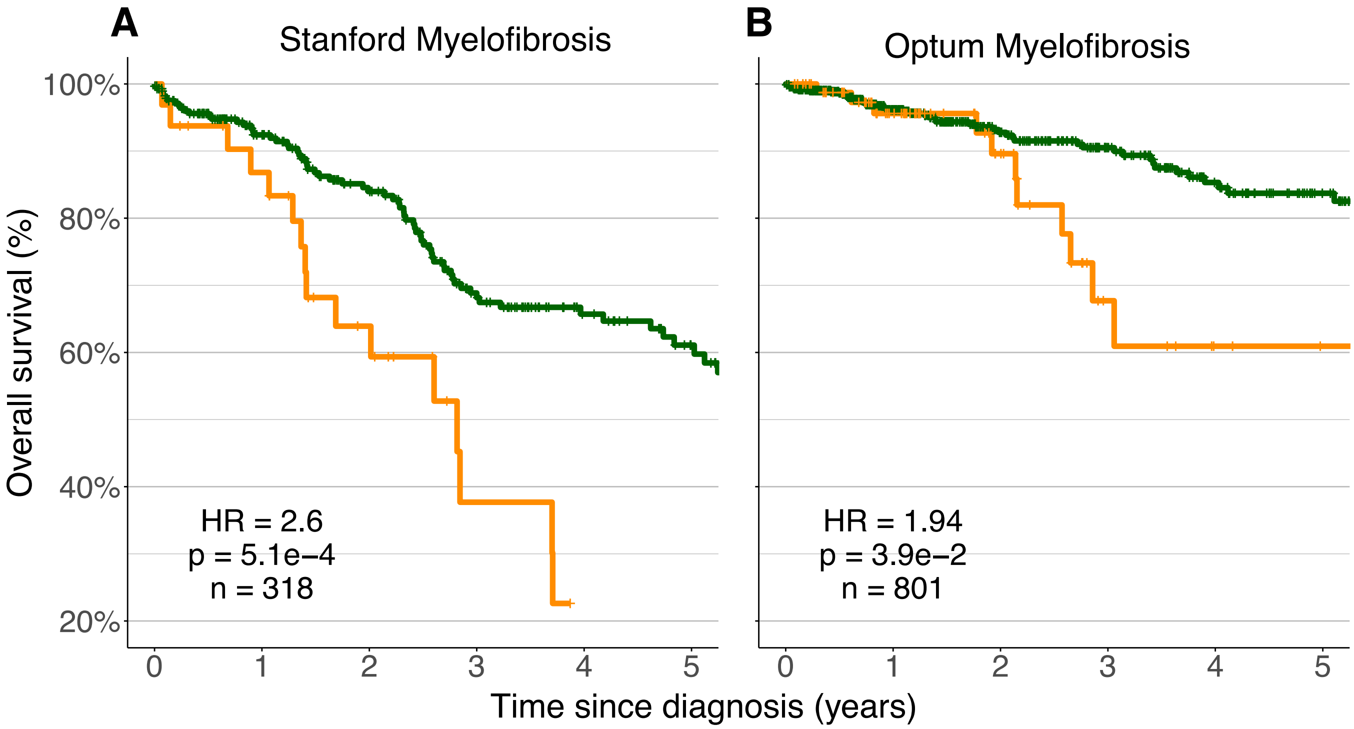

Supplementary Figure 6. Survival of myelofibrosis patients stratified by absolute monocyte count above or below the top 10% of their cohort A. Stanford (n = 318), corresponding absolute monocyte cutoff value 1.6 K/uL. B. Optum (n = 801) corresponding absolute monocyte cutoff value 1.4 K/uL.

Tables

Supplementary Table 1. Demographic information of patients from all fibrotic cohorts.

	COMET		Yale		Stanford					Vanderbilt					Optum					Northwestern
	Non-Progressors	Progressors	IPF patients	Controls	All	IPF	HCM	Myelofibrosis	SSc	All	IPF	HCM	Myelofibrosis	SSc	All	IPF	HCM	Myelofibrosis	SSc	SSc-ILD
Characteristic	(n = 29)	(n = 16)	(n = 15)	(n = 5)	(n = 1274)	(n = 130)	(n = 373)	(n = 318)	(n = 453)	(n = 16146)	(n = 1607)	(n = 8565)	(n = 3909)	(n = 2065)	(n = 27283)	(n = 5722)	(n = 9573)	(n = 801)	(n = 11187)	(n = 365)
Male (%)	62.1	68.7	86.6	40	42.4	66	54.4	56.3	14.8	52	51.2	62.5	47.6	17.6	36.3	47.9	53.6	52.9	14.5	17
Race (%)																				
White		-	100	100	58.3	56.9	56.8	61.6	57.6	79.7	86.7	81.9	86.4	78.1	-	-	-	-	-	73.7
Asian		-	-	-	12.5	15	13.4	9.7	12.8	1.1	0.9	0.9	1.5	2.1	-	-	-	-	-	-
Other		-	-	-	29.2	28.1	29.8	28.7	29.6	19.2	12.4	17.2	12.1	19.8	-	-	-	-	-	-
Hispanic Ethnicity		-	-	-	10.6	19	9.7	4.7	12.8	6.4	1.9	1.7	2.5	3.1	-	-	-	-	-	-
Age at diagnosis (yr) ± SD	65.5 ± 8.3	67.1 ± 7.9	69.4 ± 4.4	73.8 ± 5.5	59.7 ± 16.3	66.4 ± 11.3	56.7 ± 19.3	66.5 ± 11.1	55.2 ± 15.6	55.4	60 ± 18.3	58.4 ± 18	49.6 ± 22	50 ± 19	61 ± 16.18	72.1 ± 11.6	61.5 ± 15.8	68.1± 13	54.3 ± 15.3	49.2 ± 11.7
Death (%)	0	31.3	-	-	18.7	19.6	17.4	30.2	11.5	23.3	25.5	26.7	23.8	7.1	6.7	6.2	8.8	8.5	5.1	13.7
Age at death (yr) ± SD	-		-	-	69 ± 14	72.5 ± 11.6	70.1 ± 16.8	70.6 ± 10.9	62.4 ± 14.7	64.9	66 ± 16.1	65.5 ± 16.4	61.1 ± 18.3	60.24 ± 15.8	72.2 ± 12.8	78.9 ± 9.2	73.7 ± 12	73.3 ± 10.3	65.8 ± 13.2	-
Lung transplant (%)	-	6.25	-	-	4.3	29.4	1.3	0.3	1.1	1.2	5.7	0.8	0.5	0.3	0.87	4.1	0	0	0	-
FVC (%) ± SD	73.5 ± 13.9	72.8 ± 21.9	74.3 ± 15.6	-	-	61.8 ± 19.3	-	-	-	-	-	-	-	-	-	-	-	-	-	75.7 ± 17.5
DLCO (%) ± SD	44.9 ± 11.9	43.9 ± 15.8	40.2 ± 12.4	-	-	44.9 ± 19.3	-	-	-	-	-	-	-	-	-	-	-	-	-	59.6 ± 20.4
GAP Index ± SD	2.7 ± 0.8	2.9 ± 1.2	-	-	-	4.8 ± 1.3	-	-	-	-	-	-	-	-	-	-	-	-	-	1.7 ± 1.3

Supplementary Table 2. Demographic information of propensity matched cohorts from Optum and Stanford.

Characteristic	Stanford	Optum
Total patients	(n = 459)	(n = 5712)
Female (%)	24.8	48.7
Hispanic Ethnicity	7.4	N/A
Age at test (yr) ± SD	66.2 ± 14.4	69.7 ± 13.2
Death (%)	20.7	5.9
Age at death (yr) ± SD	75.8 ± 11.2	79.6 ± 9.8

Supplementary Table 3. Goodness of fit of propensity matched cohorts from Optum and Stanford.

Optum cohort	Means IPF Cohort	Means Control Cohort	SD Control	Mean Diff	eQQ Med	eQQ Mean	eQQ Max
Distance	0.5	0.3	0.2	0.2	0.1	0.2	0.5
total visits (n)	475.4	297.4	191.3	178.0	101.0	178.0	5432.0
unique diagnoses (n)	116.4	82.3	33.0	34.2	22.0	34.2	272.0
Age	68.1	71.8	14.3	-3.7	4.8	4.5	10.8
Sex							
Female	0.5	0.5	0.5	0.0	0.0	0.0	1.0
Male	0.5	0.5	0.5	0.0	0.0	0.0	1.0
Unknown	0.0	0.0	0.0	0.0	0.0	0.0	1.0
Observed duration (days)	664.8	553.9	470.8	110.9	113.0	119.6	1463.0

Stanford cohort	Means IPF Cohort	Means Control Cohort	SD Control	Mean Diff	eQQ Med	eQQ Mean	eQQ Max
Distance	0.0	0.0	0.0	0.0	0.0	0.0	0.0
total visits (n)	414.9	320.8	550.4	94.2	105.0	126.6	757.0
unique diagnoses (n)	34.9	29.3	46.0	5.5	8.0	8.8	54.0
Age	67.1	70.3	14.1	-3.1	4.6	4.2	10.3
Sex							
Female	0.3	0.2	0.4	0.1	0.0	0.1	1.0
Male	0.7	0.8	0.4	-0.1	0.0	0.1	1.0
Unknown	0.0	0.0	0.0	0.0	0.0	0.0	0.0
Observed duration (days)	632.1	1917.9	1736.5	1285.8	878.3	1215.3	2543.7

Supplementary Table 4. C-index of monocyte count or GAP index after adjusting for disease severity.

COMET (n = 36)	Variable	c-index
	GAP	0.58
	Monocytes (FVC-corrected)	0.64
	Monocytes (GAP-corrected)	0.68
**Stanford (n = 130)**		
	GAP	0.65
	Monocytes (FVC-corrected)	0.61
	Monocytes (GAP-corrected)	0.66
**Northwestern (n = 365)**		
	GAP	0.66
	Monocytes (FVC-corrected)	0.59
	Monocytes (GAP-corrected)	0.66
